# Supplementary figures and images for: A genome-wide enrichment screen identifies NUMA1-loss as a resistance mechanism against mitotic cell-death induced by BMI1 inhibition
Source: PLoS One. 2020 Apr 28;15(4):e0227592. doi: 10.1371/journal.pone.0227592 (PMC7188281; doi:10.1371/journal.pone.0227592)

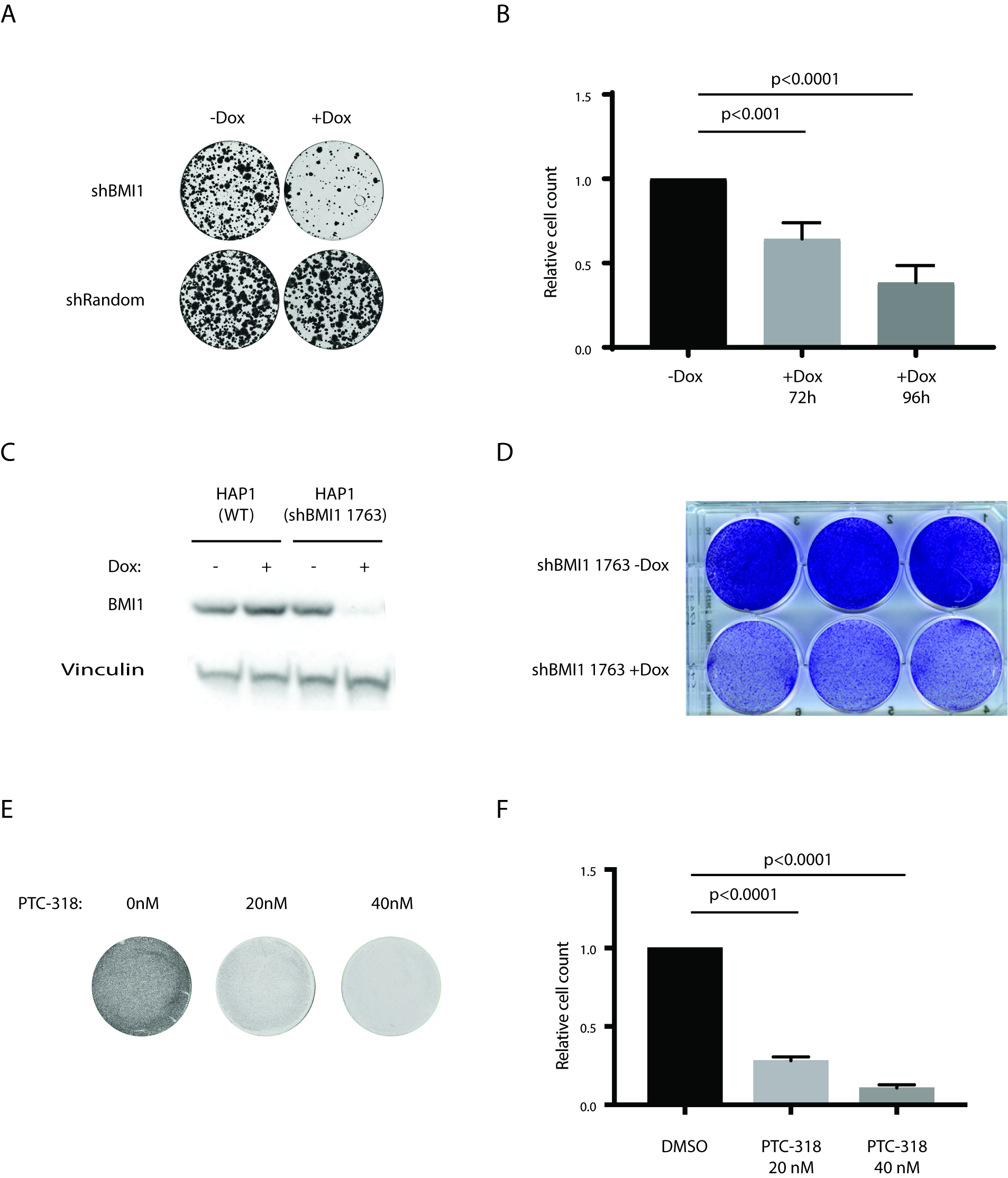

Supplement: S1 Fig — (A) Cell survival upon BMI1 knockdown was confirmed in HAP1 cells transduced with shBMI1 through a colony formation assay one week after plating HAP1 cells transduced with either shRandom or shBMI1 and treated with doxycycline or (B) through relative cell counts of HAP1 cells transduced with shBMI1 treated with doxycycline for 72, and 96 hours after plating or without doxycycline (-Dox). (C and D) Validation of BMI1 knockdown-induced cytotoxicity using a different shRNA (shBMI1 1763). (C) BMI1 protein expression levels of wildtype HAP1 and HAP1 transduced with shBMI1 1763 with or without doxycycline treatment (+Dox or -Dox, respectively) for 96 hours. (D) Colony formation assay showing shBMI1 1763 transduced HAP1 cells 96 hours with or without doxycycline. Technical replicates. (E) Cell survival of HAP1 cells treated with PTC-318 through colony formation assay one week after treatment with DMSO (0.1%) or PTC-318 (20 or 40 nM) and (F) relative cell counts 48 hours after treatment with DMSO (0.1%) or PTC-318 (20 or 40 nM). Error bars represent SD. Student’s t-test was performed for statistical testing. Error bars for both graphs represent SD (n = 3). (TIF) [file pone.0227592.s001.tif]

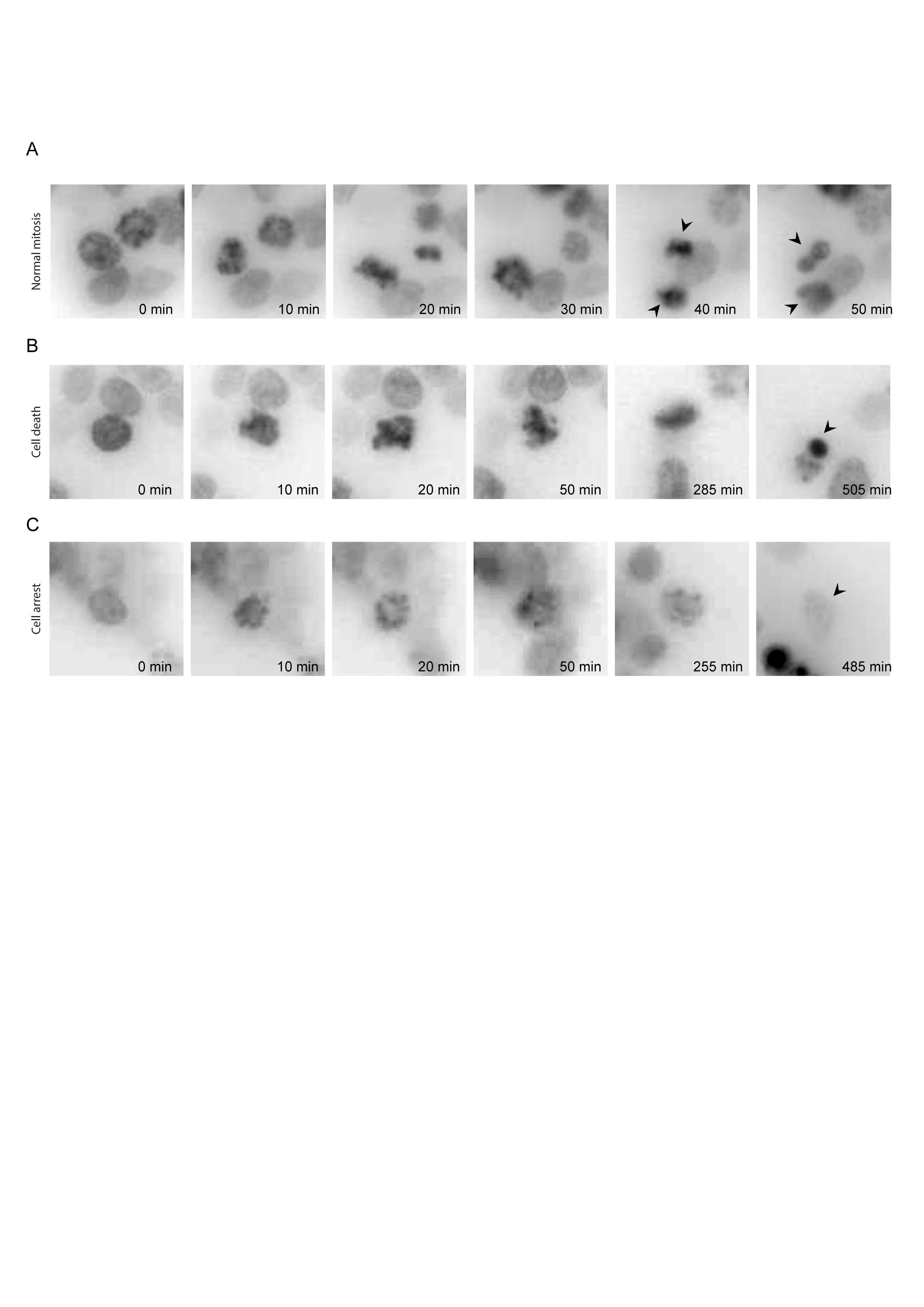

Supplement: S2 Fig — (A) HAP1 cells undergoing a normal mitosis with chromosome segregation (highlighted by the arrowheads). B) HAP1 cell dying after a prolonged mitotic arrest. Arrowheads point to the dying cell. C) HAP1 cell arrested in mitosis followed by slippage to interphase without DNA segregation (arrowhead). Time 0 min corresponds to nuclear envelope breakdown. (TIF) [file pone.0227592.s002.tif]

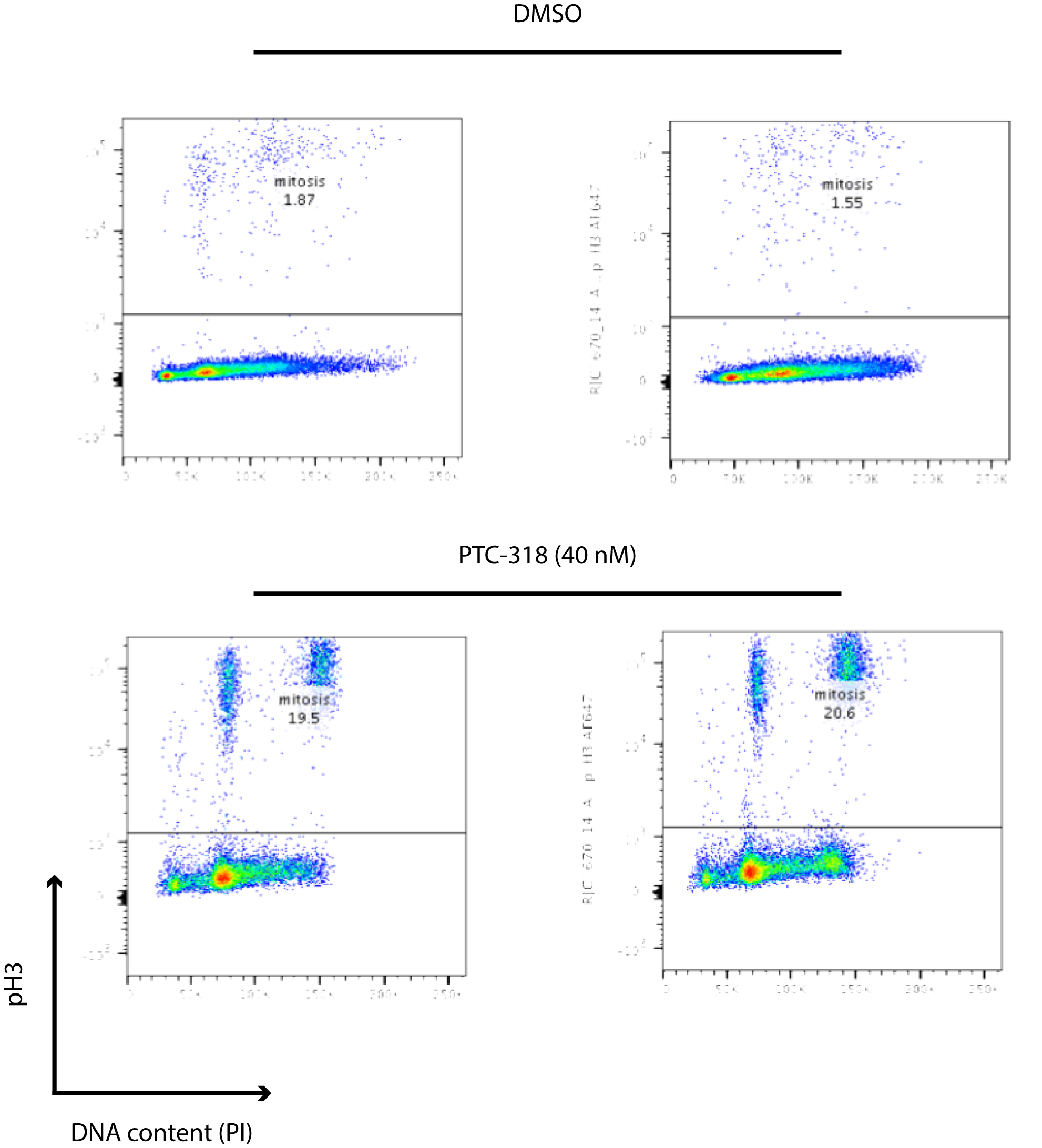

Supplement: S3 Fig — Flow plot comparing the proportion of cells in mitosis between HAP1 cells treated with DMSO and PTC-318. Technical replicates. (TIF) [file pone.0227592.s003.tif]

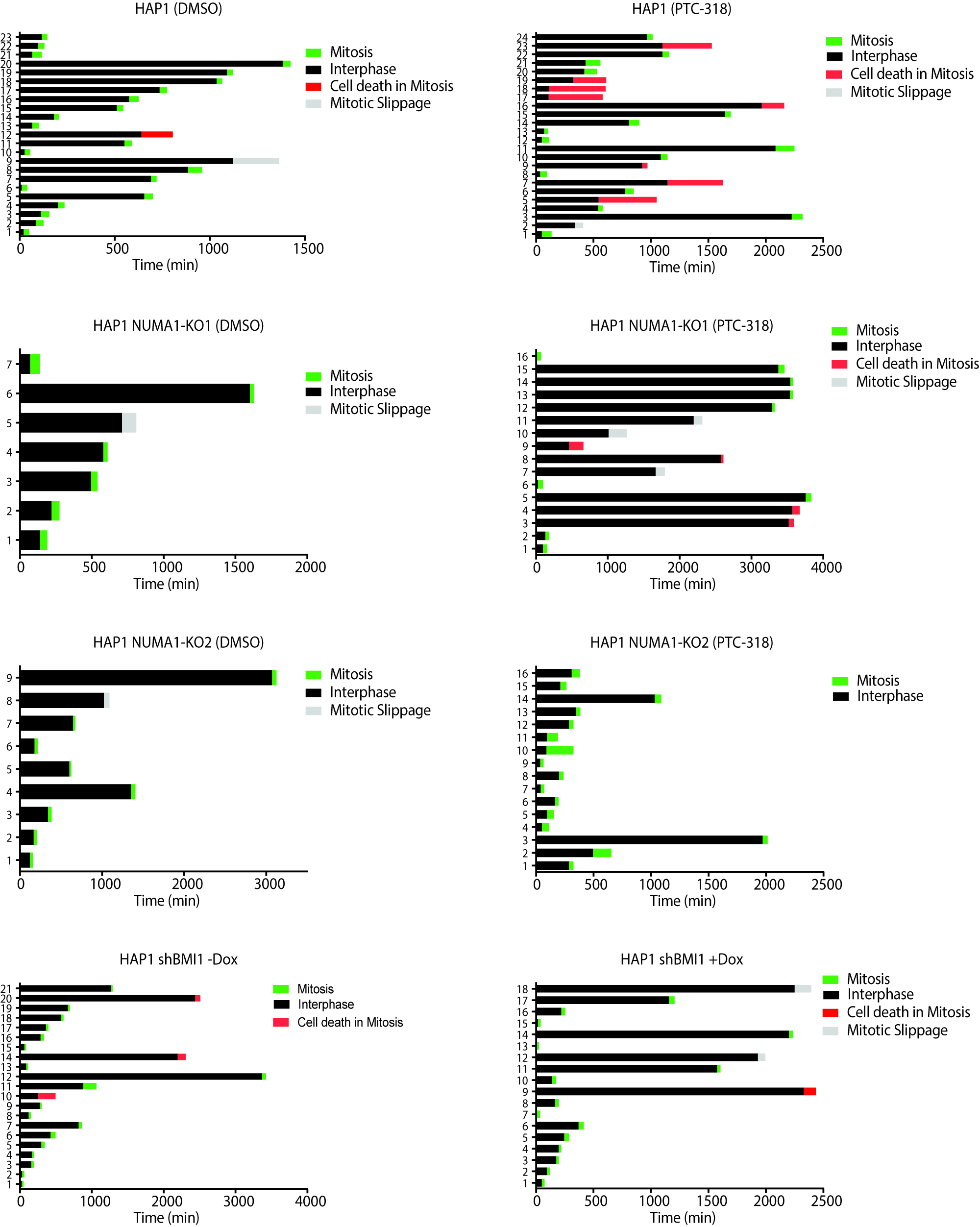

Supplement: S4 Fig — Quantification of live-cell imaging data showing the times of individual cells. Upper three rows show cells treated with either DMSO (0.1%) or PTC-318 (20 nM) while the lower row shows HAP1 cells transduced with shBMI1 untreated (-Dox) or treated (+Dox) with doxycycline. The respective y-axes depict the individual clones. (TIF) [file pone.0227592.s004.tif]

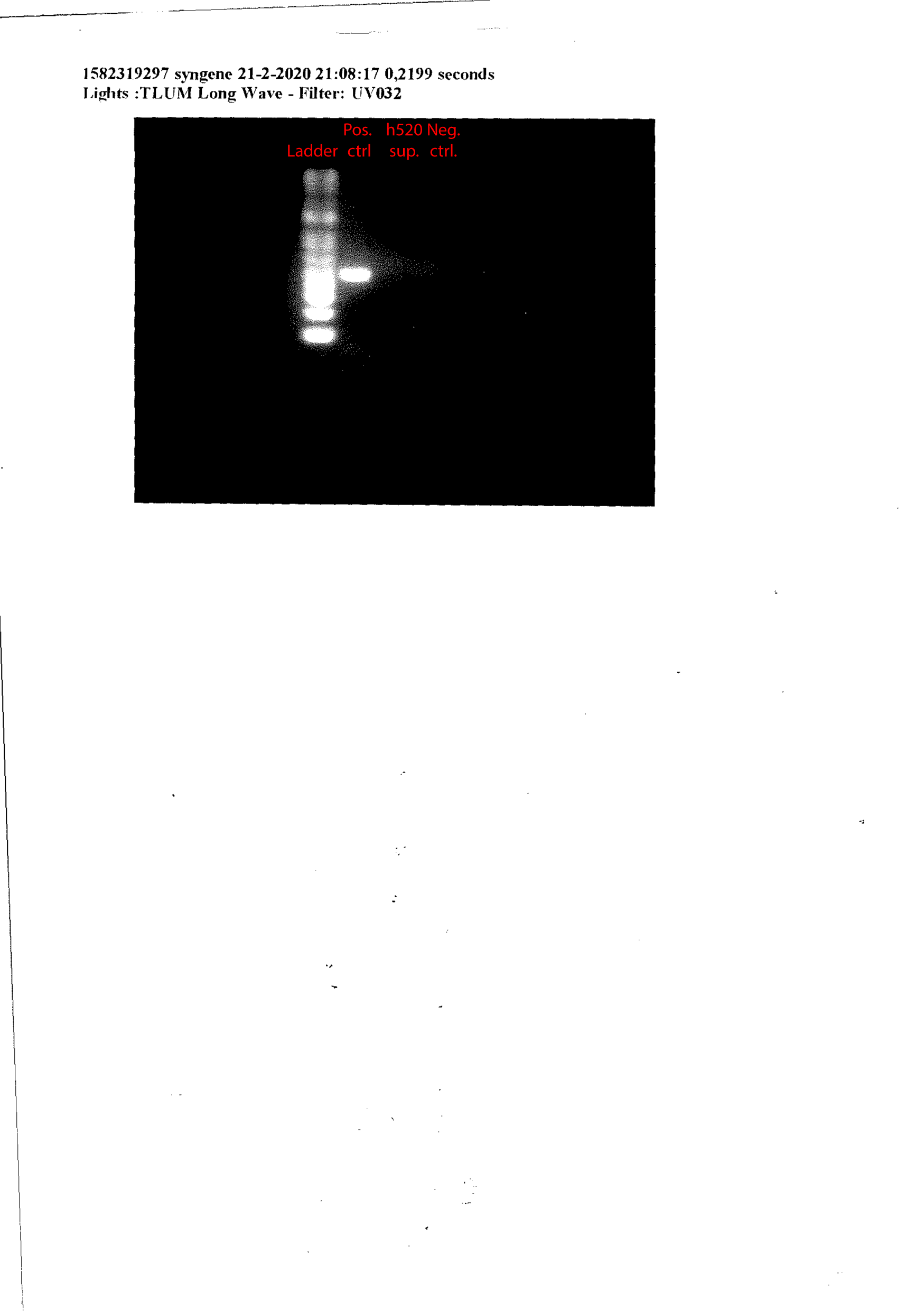

Supplement: S5 Fig — (TIF) [file pone.0227592.s005.tif]

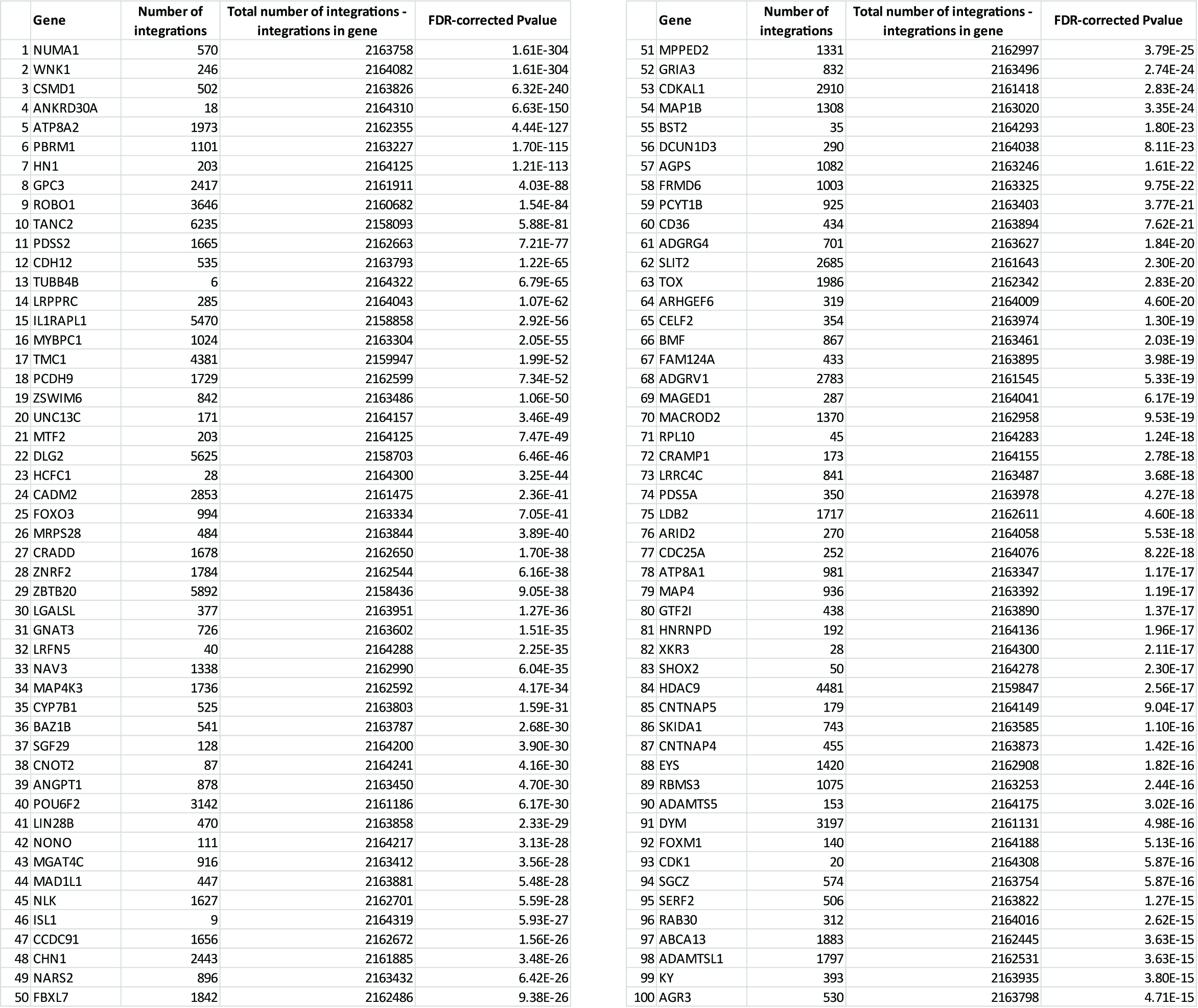

Supplement: S1 Table — (TIF) [file pone.0227592.s006.tif]
