# Supplementary material for: A genome-wide enrichment screen identifies NUMA1-loss as a resistance mechanism against mitotic cell-death induced by BMI1 inhibition
Source: PLoS One. 2020 Apr 28;15(4):e0227592. doi: 10.1371/journal.pone.0227592 (PMC7188281; doi:10.1371/journal.pone.0227592)

Fig 1B

	L1	L2	L3	L4	L5	L6
Cell line	Marker	HAP1	HAP1	HAP1	HAP1	HAP1
Genetic background		shBMI1	shBMI1	shBMI1	shBMI1	shBMI1
Treatment		-	Dox 24h	Dox 48h	Dox 72h	Dox 96h

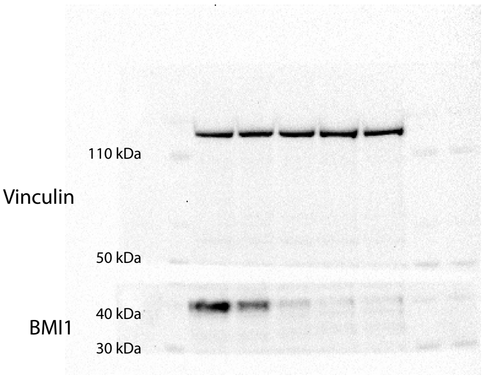

Fig 1E

	L1	L2	L3	L4	L5	L6	L7	L8	L9	L10	L11	L12
Cell line	Marker	HAP1	HAP1	HAP1	HAP1	HAP1	HAP1	Marker	X	X	X	Marker
Genetic background		WT	WT	WT	WT	WT	WT					
Treatment		DMSO	PTC-318 10 nM	PTC-318 20 nM	PTC-318 30 nM	PTC-318 40 nM	PTC-318 50 nM					

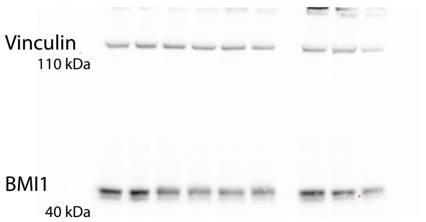

Fig 2A

	L1	L2	L3	L4	L5	L6	L7	L8	L9	L10
Cell line	Marker	HAP1	HAP1	X	X	Marker	Marker	X	X	Marker
Genetic background		WT	FUGW-BMI1							
Treatment		-	-							

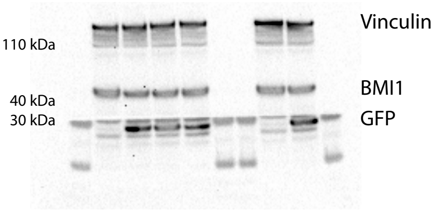

Fig 3A

	L1	L2	L3	L4	L5	L6	L7	L8	L9	L10	L11	L12
Cell line	Marker	HAP1	HAP1	HAP1	X	EMPTY	EMPTY	X	X	X	X	Marker
Backgorund 1		WT	NUMA-KO	NUMA-KO								
Background 2		WT	shBMI1	shBMI1								
Clone			clone 1	clone 2								

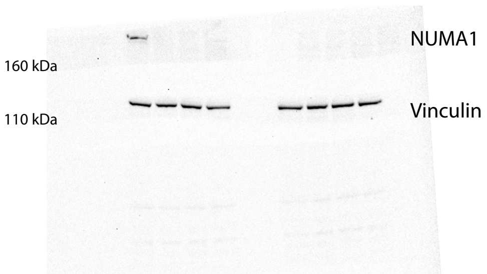

Fig 3D

	L1	L2	L3	L4	L5	L6	L7	L8	L9	L10
Cell line	Marker	HAP1	HAP1	EMPTY	HAP1	HAP1	EMPTY	HAP1	HAP1	Marker
Backgorund		WT	WT		NUMA1-KO 1	NUMA1-KO 1		NUMA1-KO 2	NUMA1-KO 2	
Treamtment		DMSO	PTC-318 (40 nM)		DMSO	PTC-318 (40 nM)		DMSO	PTC-318 (40 nM)	

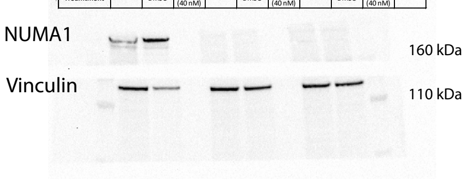

Fig 3G (blot 1 of 2)

	L1	L2	L3	L4	L5	L6	L7	L8	L9	L10	L11	L12	L13
Cell line	Marker	X	HAP1	HAP1	HAP1	HAP1	HAP1	HAP1	HAP1	HAP1	HAP1	HAP1	Marker
Transfection			BMI1-KO	BMI1-KO	BMI1-KO	BMI1-KO	BMI1-KO	BMI1-KO	BMI1-KO	BMI1-KO	BMI1-KO	BMI1-KO	
Clone			1	2	3	4	5	6	7	8	9	10	

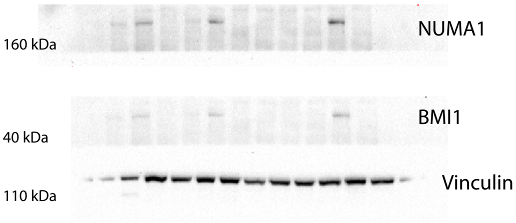

Fig 3G (blot 2 of 2)

	L1	L2	L3	L4	L5	L6	L7	L8	L9	L10	L11	L12	L13
Cell line	Marker	X	HAP1	HAP1	HAP1	HAP1	HAP1	HAP1	HAP1	HAP1	HAP1	HAP1	Marker
Transfection			BMI1-KO	BMI1-KO	BMI1-KO	BMI1-KO	BMI1-KO	BMI1-KO	BMI1-KO	BMI1-KO	BMI1-KO	BMI1-KO	
Clone			11	12	13	14	15	16	17	18	19	20	

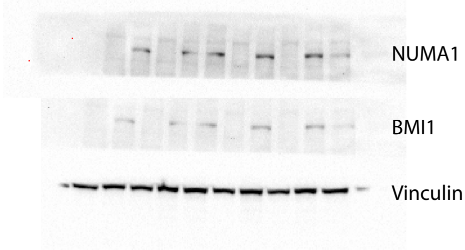

Fig 4D

	L1	L2	L3	L4	L5	L6	L7	L8	L9
Cell line	Marker	Marker	HAP1	HAP1	HAP1	HAP1	HAP1	HAP1	Marker
Treatment			DMSO	PTC-318 (40 nM)	PTC-318 (40 nM)	PTC-318 (40 nM)	PTC-318 (40 nM)	PTC-318 (40 nM)	
Treatment time			-	2h	4h	6h	8h	24h	

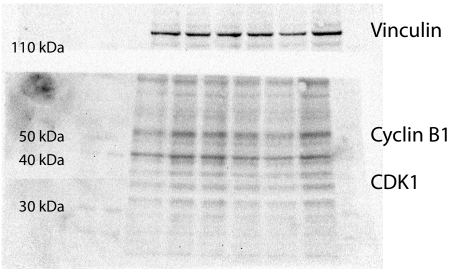

Fig 5E

	L1	L2	L3	L4	L5	L6	L7	L8
Cell line	Marker	h520	h520	h520	h520	h520	h520	Marker
Treatment		BMI1-KO	BMI1-KO	BMI1-KO	BMI1-KO	BMI1-KO	BMI1-KO	
Clone		clone 1	clone 2	clone 3	clone 4	clone 5	clone 6	

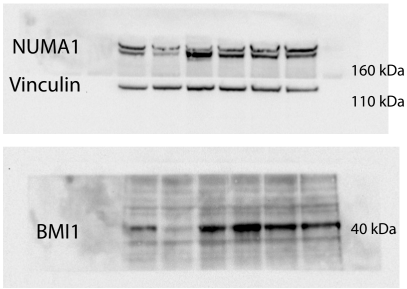

S1 Fig C

	L1	L2	L3	L4	L5	L6	L7	L8
Cell line	Marker	HAP1	HAP1	HAP1	HAP1	X	X	Marker
Background		shBMI1 1763	shBMI1 1763	WT	WT			
Treatment		+Dox	-Dox	+Dox	-Dox			

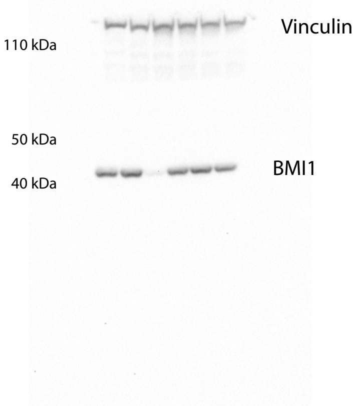

Supplement: S1 Raw Images — (PDF) [file pone.0227592.s007.pdf]
